# Data-Driven Hypothesis Generation in Clinical Research: What We Learned from a Human Subject Study?

**DOI:** 10.18103/mra.v12i2.5132

**Published:** 2024-02-28

**Authors:** Xia Jing, James J. Cimino, Vimla L. Patel, Yuchun Zhou, Jay H. Shubrook, Chang Liu, Sonsoles De Lacalle

**Affiliations:** 1. Department of Public Health Sciences, College of Behavioral, Social and Health Sciences, Clemson University, Clemson, SC; 2. Informatics Institute, School of Medicine, University of Alabama, Birmingham, Birmingham, AL; 3. Cognitive Studies in Medicine and Public Health, The New York Academy of Medicine, New York City, NY; 4. Department of Educational Studies, Patton College of Education, Ohio University, Athens, OH; 5. Department of Clinical Sciences and Community Health, Touro University California College of Osteopathic Medicine, Vallejo, CA; 6. Department of Electrical Engineering and Computer Science, Russ College of Engineering and Technology, Ohio University, Athens, OH; 7. Department of Health Science, California State University Channel Islands, Camarillo, CA

**Keywords:** Clinical research, scientific hypothesis generation, visualization, data-driven hypothesis generation, medical informatics, translational research

## Abstract

Hypothesis generation is an early and critical step in any hypothesis-driven clinical research project. Because it is not yet a well-understood cognitive process, the need to improve the process goes unrecognized. Without an impactful hypothesis, the significance of any research project can be questionable, regardless of the rigor or diligence applied in other steps of the study, e.g., study design, data collection, and result analysis. In this perspective article, the authors provide a literature review on the following topics first: scientific thinking, reasoning, medical reasoning, literature-based discovery, and a field study to explore scientific thinking and discovery. Over the years, scientific thinking has shown excellent progress in cognitive science and its applied areas: education, medicine, and biomedical research. However, a review of the literature reveals the lack of original studies on hypothesis generation in clinical research. The authors then summarize their first human participant study exploring data-driven hypothesis generation by clinical researchers in a simulated setting. The results indicate that a secondary data analytical tool, VIADS—a visual interactive analytic tool for filtering, summarizing, and visualizing large health data sets coded with hierarchical terminologies, can shorten the time participants need, on average, to generate a hypothesis and also requires fewer cognitive events to generate each hypothesis. As a counterpoint, this exploration also indicates that the quality ratings of the hypotheses thus generated carry significantly lower ratings for feasibility when applying VIADS. Despite its small scale, the study confirmed the feasibility of conducting a human participant study directly to explore the hypothesis generation process in clinical research. This study provides supporting evidence to conduct a larger-scale study with a specifically designed tool to facilitate the hypothesis-generation process among inexperienced clinical researchers. A larger study could provide generalizable evidence, which in turn can potentially improve clinical research productivity and overall clinical research enterprise.

## 1 Introduction

A hypothesis is an educated guess about the relationships among several variables ^1,2^. Hypothesis generation occurs at the very early stage of the lifecycle of a research project ^1,3–5^. Typically, after hypothesis generation, study design, data collection, data analysis, results and conclusion dissemination occur sequentially ^1,4^ Without an impactful hypothesis, no matter how rigorous the study design, how careful the experimental execution, or how detailed the analysis of results, the impact of a research project will be limited. Despite the importance of hypothesis generation in scientific studies, the cognitive process of hypothesis generation has not yet been well understood. Our group has conducted a data-driven hypothesis generation study with clinical researchers to explore the process in the clinical research context ^6–10^. We developed a visual interactive analytical tool for filtering, summarizing, and visualizing large health data sets coded with hierarchical terminologies—VIADS ^11–16^, and we compared the hypothesis generation processes among clinical researchers when they used VIADS and any other analytical tools, such as Excel, SPSS, R. The original study protocol ^10^ and detailed individual aspects of the study results have been published separately, including usability, utility, hypothesis measure instruments, cognitive events ^6–9^. In this perspective paper, we aim to (1) provide a literature review on the intersectional context of scientific thinking, reasoning, discovery, medical reasoning, and literature-based discovery in clinical research that serves as the background of our study and (2) elaborate on our study, its methods and results, its significance, and its roles within the clinical research context.

Scientific hypothesis generation, which aims at developing research projects to pursue later, can be categorized into at least two broad groups. The first category typically originates from observing expected or unexpected phenomena during wet-lab experiments or other types of data collection, such as in traditional chemical or biological studies. The second category typically originates from secondary data analysis, usually called data-driven hypothesis generation; this category is often used in epidemiology, psychology, and informatics studies. In hypothesis-driven research, and compared with predictive research, a hypothesis has a central role in the project and its lifecycle ^17^. Our study focuses on the second category, specifically in a clinical research context.

In daily life, hypotheses are used constantly, and mostly unconsciously. For example, while driving on a busy highway, the decision to change lanes is based on hypotheses related to prior experiences, the surrounding vehicles’ behavior, and relative speeds and distances among all these vehicles. Most drivers can maneuver successfully without explicitly articulating which step is hypothesis generation and which is hypothesis testing. This process occurs very rapidly and is usually not accomplished consciously. Many hypothesis generations refer to everyday hypotheses. However, the focus in our study is on *scientific* hypothesis generation. The hypothesis we focus on will be used in sequential scientific research studies to prove or disprove the hypothesis to move the boundaries of science.

Scientific hypothesis generation is part of scientific thinking, which also includes scientific reasoning, medical reasoning, and problem-solving ^18–20^. However, they are not identical to one another. Scientific thinking is a broader concept, and most often requires reasoning and problem-solving. While, hypothesis generation also requires reasoning capability, there are several differences between hypothesis generation, scientific reasoning and problem-solving. First, hypothesis generation is an exploration process to look for a problem to focus on, whereas scientific reasoning and problem-solving are mostly used when one already has a problem, puzzle, or medical case in hand and is trying to solve the issue. Second, the process of hypothesis generation is largely exploratory, without fixed answers, whereas scientific reasoning and problem-solving usually have one or several correct answers to reach. Third, hypothesis generation uses more divergent thinking, whereas scientific reasoning and problem-solving use more convergent thinking ^19^, which indicates that the underlying mechanisms used by these cognitive processes may be different. Many successful studies have explored scientific reasoning in educational settings to solve puzzles or learn new functions of an existing tool ^21–23^, as well as in medical settings for diagnosis, or differential diagnosis issues ^24–26^. However, scientific hypothesis generation with human participants is rare in the literature.

Although hypothesis generation is an early step in scientific studies and research projects ^1^ and its critical role has been broadly recognized ^27–29^, few studies have focused on understanding the principles or exploring the mechanisms of the process. There have been studies in literature mining ^30^, the ABC model ^31–33^, and automatic systems to generate hypotheses ^34–37^. These studies explored the scientific hypothesis generation and established the critical foundation for further research, especially the ABC model, which has guided a significant portion of studies in this area for decades. However, extremely few studies have included human participants’ evaluations in these studies. Considering the complex nature of the hypothesis generation process, studying how humans generate hypotheses has unique advantages for better understanding the process and underlying mechanisms and improving it.

The rest of the article is organized into the following sections: a literature review to set the background for our study, a summary of the methods and results of our study, discussions about interpreting the results and reflecting on the study, and conclusion. Our study shows VIADS was perceived as a helpful tool in facilitating hypothesis generation by clinical researchers; among inexperienced clinical researchers, participants in the VIADS group used significantly shorter time and used significantly fewer cognitive events to generate each hypothesis on average; however, hypotheses generated by participants in the VIADS group received significantly lower ratings in feasibility. Through the study there are much more questions identified than answered regarding to hypothesis generation in clinical research and more research is needed in this field.

## 2 Literature Review of Other Studies

Our study focuses on scientific hypothesis generation, which has not been an established field by itself, i.e., very few studies focus on scientific hypothesis generation per se. However, there are studies in relevant fields. Therefore, to acknowledge the existing relevant work, we explored and reviewed the literature in the following topics: scientific thinking and reasoning, medical reasoning, literature-based discovery, and field study on scientific thinking and discovery. Under each topic, we introduce a literature review of the topic and what we can learn from these studies. A comprehensive summary of the four topics concludes the literature review section. We then introduce our study objectives before summarizing the methods and results of our study.

### 2.1 SCIENTIFIC THINKING AND REASONING

#### 2.1.1 Literature Overview and Main Findings

**Figure 1:**
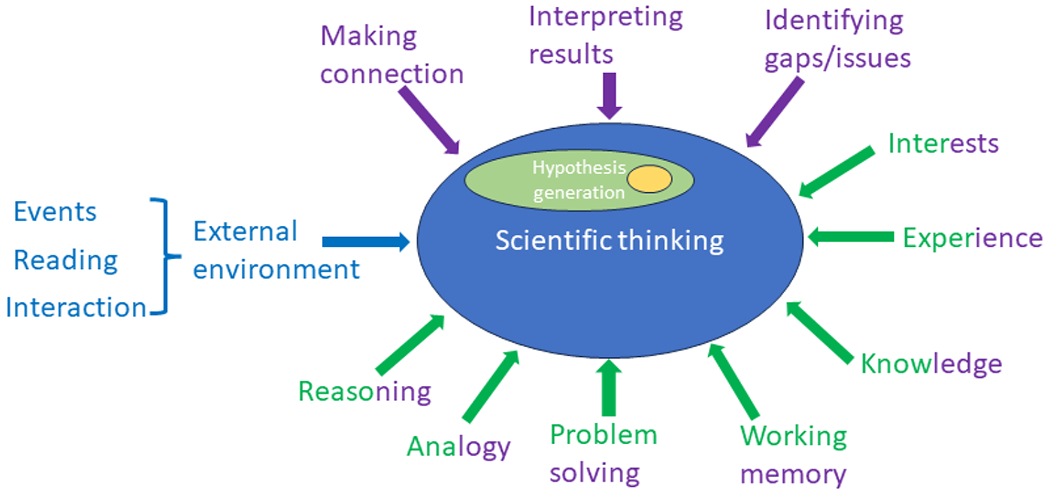
Conceptual model showing the relationships among scientific thinking, hypothesis generation, and their contributing capabilities and attributes (purple, domain-related cognitive capabilities or obtained attributes; green, generic cognitive capabilities or obtained attributes; gold, our focus)

Scientific thinking refers to the cognitive processes used during scientific-related activities ^18,19^. An elaboration of scientific-related activities can include at least the following events: hypothesis generation, formulating research questions, designing the study, collecting data, analyzing data, and writing and publishing results, which is a typical lifecycle of a scientific study ^1,3,4^. The thinking involved in each of these events can be categorized as scientific thinking. Although scientific thinking and reasoning are often used together, reasoning is one of the cognitive capabilities, along with analogy, decision-making, problem-solving, and working memory ^38,39^, all of which are critical and necessary to scientific thinking. By contrast, Kuhn et al., considered scientific thinking to be logical thinking, problem-solving, and induction ^40^. Other contributing factors of scientific thinking include prior knowledge, memory, data generated from experiments, accidental events, and systematically generated evidence ^20^. Figure 1 shows a conceptual framework of scientific thinking, its supporting and necessary cognitive capacities and attributes, and their primary relationships. As shown in Figure 1, the focus of our study is a small subset of hypothesis generation, which is a subset of scientific thinking.

Scientific thinking has the potential to exert a substantial impact on scientific education and scientific discoveries. Many researchers have emphasized the coordination of theories and evidence in scientific education ^40^, and others have focused on conceptual change, especially during paradigm shifts within scientific education and scientific discoveries ^41^. Klahr proposed two spaces of problems that characterize scientific reasoning—one related to hypothesis and the other having to do with evidence ^20,42,43^. Klahr proposed that the framework could explain hypothesis generation, experiment design, hypothesis evaluation, and the interactions among these processes ^42^. However, the hypotheses used in above mentioned contexts are not the hypotheses used in research settings to develop potential research projects; rather, the hypotheses Klahr referred to are the ones used to solve puzzles during experimentation. We purposefully distinguish the two types of hypotheses because of the potentially different mechanisms underneath during hypothesis generation. In fact, the generation of hypotheses for research projects, especially data-driven hypotheses, most likely use divergent thinking with multiple possible correct answers, whereas the generation of hypotheses to solve a puzzle likely uses convergent thinking with a limited number of correct answers ^19^.

Within scientific thinking, some researchers have also studied hypothesis generation. Thomas et al. ^44^ proposed a human judgment framework to generate hypotheses and explained hypothesis testing and human judgment. Their study, however, was focused on human judgment, decision-making, and the hypotheses generated in order to do so. Later, Sprenger et al. ^45^ demonstrated that divided attention could lead to a reduced number of alternative hypotheses generated or errors, bias, or limitations during information retrieval, and further lead to errors or bias in judgment by using the same framework. The results were also confirmed by Dasgupta et al. ^46^ with additional experiments and simulations. Donnelly et al. ^47^ demonstrated that 7–10 tasks can be reliably used to test hypotheses in clinical problem-solving with medical students as participants. Although the contexts of these studies are not particularly relevant to clinical research, the results are still helpful and informative in our study design. Alison et al. ^48^ demonstrated that time pressure reduced the number of hypotheses generated in a police investigation context. Merrifield and Erickson ^49^ showed that statistics enhanced overall judgment and the experience level of participants during hypothesis generation within a simulated nuclear attack scenario with the Reserve Officer Training Corps—ROTC students as participants.

Furthermore, in order to measure creativity, which is a critical attribute of a scientific hypothesis, Dumas and Dunbar ^21^ used semantic analysis to measure new ideas with a psychometric test: The Use of Objects Task by undergraduates. They demonstrated that semantic analysis can be used as an objective measure of the originality of ideas, although the originality and ideas in their study are not in a scientific research context but in a more generic English language context. Kerne et al. ^50^ also attempted to measure new ideas for originality. Similarly, their study—which was not placed within a scientific research context—used open-ended questions with grading criteria in an information discovery context.

#### 2.1.2 What We Can Learn from the Literature

Scientific reasoning is an important cognitive capability for conducting scientific thinking and hypothesis generation; however, scientific reasoning is not identical to scientific thinking or hypothesis generation. Using a puzzle or enumerating correct answers is an excellent way to study reasoning and compare results consistently in a scientific study; however, it is slightly far from measuring the real scientific hypothesis generation process or scientific hypothesis quality within research settings. Using scientific reasoning alone to represent scientific thinking somewhat simplifies the scientific thinking process. The examining of the literature indicates the scientific hypothesis generation process within the scientific research context is not the focus of most studies. We do, however, acknowledge that the literature and previous experiments provide tangible examples of comparison and task setting for human participants’ studies of scientific hypothesis generation in a clinical research context.

### 2.2 MEDICAL REASONING

#### 2.2.1 Literature Overview and Main Findings

Within scientific reasoning, medical reasoning has been actively explored by many researchers in the past several decades, perhaps for two reasons. One, because medical reasoning can be critical to improve medical education and practice; and two, because medical reasoning provides a scenario that is closer to real-world reasoning, often with limited, incomplete, and sometimes inaccurate information. In the medical realm, sometimes the results cannot be verified easily or quickly; very often, the results are more complicated than a binary result. Patel et al., a pioneer group in this field, verified the relationship between forward or data-driven reasoning and accurate diagnosis among cardiologists, psychiatrists, and surgeons ^51^. Several original studies from Patel’s group explored hypothesis generation and testing in medical diagnostic tasks and showed differences between medical novices and experts in developing their diagnoses ^25,26,52–54^. The experts used more data-driven reasoning, whereas the novices used more hypothesis-driven reasoning; the experts used their more developed and a more knowledge-rich structure, whereas the novices used their knowledge-lean structure during the reasoning processes. Furthermore, experts usually skipped steps in their reasoning process ^26,51–54^, showing that they do not explicate every step in the reasoning process. Through protocol analysis ^55,56^, think-aloud techniques have been used in many studies to make the implicit reasoning processes more explicit ^57–61^. Similar methods have been applied as a relatively mature technology for evaluating the usability of health information technology-related systems ^58,62,63^. For more details readers are referred to two book chapters ^19,64^ in the thinking and reasoning textbooks.

#### 2.2.2 What We Can Learn from the Literature

The studies in medical reasoning provide helpful insights, particularly regarding our study design: these studies inspired us separating inexperienced and experienced clinical researchers, seeking to elucidate whether there are different processes in generating scientific hypotheses in those two groups. These studies also suggested think-aloud protocol can be used to decipher the process. While we acknowledge that clinical practice and clinical research have slight differences regarding urgency, especially during the hypothesis development and verification stages, the former is usually within an extremely limited time frame, but the latter is not under similar time constraints. These differences could result in significantly different outcomes during the applications in the two related but different contexts.

### 2.3 LITERATURE-BASED DISCOVERY

#### 2.3.1 Literature Overview and Main Findings

Don Swanson’s ABC model was published in 1986 ^31,32^, which initiated the research field of using publicly available information and literature to reveal existing but unknown relationships between concepts. Those newly revealed relationships could then serve as the initial hypotheses or components of scientific hypotheses for future studies. This type of study was described as literature-based discovery or literature mining ^30^. Several researchers developed systems to reveal existing but unknown relationships for hypotheses generation. Arrowsmith is an example that used the ABC model to conduct literature mining ^33,65^. SemRep ^66^ is another example of literature based discovery utilized ABC model. It is a natural language processing system that extracts semantic relationships from biomedical literature collected in PubMed ^67^.

Sam Henry, et al. ^30^ published a literature review with a comprehensive analysis of the existing literature and research on literature-based discovery. The literature review covers the following aspects of literature-based discovery: (a) language processing operation, e.g., term removal or representations, (b) different literature discovery models, e.g., co-occurrence, semantic, distributional, and user interaction, (c) components of the systems, (d) evaluations, (e) application areas, and (f) challenges. In the literature review, Henry et al. distinguished between open discovery and close discovery ^30^. The open discovery is similar to the scientific hypothesis generation that we focused on in our study; the close discovery is similar to the scenarios used in scientific reasoning experiments ^42^.

Within the domain of literature-based discovery, some researchers have focused on the basic units of a sentence, that is, entities and relationships, how to identify them, and how to improve the performance of the identifications. The application of these techniques to the clinical literature have resulted in a number of studies focused on entity identification ^68,69^, and others have focused on relationship identifications ^66,70–73^ and temporal pairs of terms identification ^74^; other literature has focused on similarity measurements ^75–77^, which can be used to categorize the identified entities or relationships. Some studies have also attempted scientific discoveries by identifying outlier literature ^78,79^ or missing concepts ^80^ to facilitate literature-based discovery. Finally, some researchers have built systems to conduct similar tasks and study users’ information-seeking behavior ^81^ while using the system. SemRep ^66^, RajoLink ^82^, Spark ^83^, EpiphaNet ^84^, and the framework based on information foraging theory ^85^ are a few examples of such efforts.

In addition to literature-based discovery, some researchers have attempted to generate hypotheses automatically, mostly by leveraging scientific literature mining ^36^, biomedical literature ^34,37,86–88^, and semantic web technology and ontology ^35^. In addition to automatic hypothesis generation systems, researchers have attempted to validate hypotheses ^89^, evaluate hypotheses ^90^ on specific topics, such as galactose metabolism in *Saccharomyces cerevisiae*, and conduct more basic studies related to hypotheses, such as representation ^91^ of hypotheses and using graph theory and logical modeling of biomedical networks to generate hypotheses ^92^. Despite the example systems, researchers acknowledged that completely automatic hypothesis generation remains unrealistic and hypothesis generation remains human-centered ^35–37,88,90,91,93^.

Large language models—LLMs have recently dominated scientific, technical, and other headlines. Researchers have also attempted to test whether LLM can generate hypotheses automatically ^94–95^. The results showed that although describing the structure of scientific knowledge appeared effective ^95^, the error rates were still high. Noticeably, hallucination has been identified as a major concern in the applications of LLM in the generic or biomedical fields ^96–98^, not to mention the ethical concerns of using LLM in healthcare ^99^. In addition, the reasoning capacity of LLM in a clinical research context remains unknown, although experiments have shown that LLM can improve the performance of inductive reasoning, but with low levels of accuracy, approximately 27.5% ^94^. Hallucinations can be perceived as an appealing attribute during human–machine interactions in social settings. However, such shortcomings can be fatal flaws for more formal use scenarios of LLM, such as applications in scientific research, in which precise facts and meticulous logic are necessary and commonly used to conduct inference and reasoning.

#### 2.3.2 What We Can Learn from the Literature

As described above, there is active exploration of different methodologies and systems to reveal existing but unknown relationships, which can be used to generate scientific hypotheses. However, in such processes, not necessarily something new was created or generated from existent substances; rather, something unknown was revealed. Although the ABC model is impactful and has influenced many such studies, the paradigm it represents is a commonly used type of hypothesis, not all possible hypotheses in a scientific research context. Meanwhile, the existing literature mining systems with user interfaces lack systematically human-participated evaluation studies.

Although it has been demonstrated that LLM can generate fluent English, LLM may not be best suited for generating new ideas or scientific hypotheses for research projects, because it is not precisely contextualized. This is a substantial concern in using LLM in more rigorous settings, such as study design. LLM seems to provide promises, possibilities, and hopes for scientific hypothesis generation or other aspects of scientific research; however, it is not yet at a stage that can be reliably used or even tested systematically with robust metrics and thorough requirements. A completely automatic system to generate research hypotheses is unrealistic yet; humans have to be in the loop and at the center to ***create*** new ideas, perhaps by leveraging existing technologies, such as LLM, to perform better than humans alone or technology alone.

### 2.4 FIELD STUDY OF SCIENTIFIC THINKING AND DISCOVERY

#### 2.4.1 Literature overview and main findings

In vivo cognitive studies have been used to describe the cognitive investigations conducted in the real world versus those experiments conducted in a laboratory setting, which have been named in vitro cognitive studies ^18,19^. In vitro settings provide several advantages for scientific research, such as better control of the conditions and comparable groups. In vitro settings are especially suitable for identifying individual factors for specific mechanisms. However, they are not free from limitations ^100^ and not all in vitro settings can reflect or mimic the real-world experience completely ^101^. By contrast, in vivo cognitive studies have many advantages. For example, Dunbar’s group conducted an in vivo cognitive study to examine scientific thinking and discovery processes in real time and in the natural environments. They chose four laboratories from six candidate laboratories in a US university, all four conducting highly innovative basic biomedical research, with recognized reputations and excellent track records in their fields. Dunbar interviewed 19 scientists in these four laboratories, participated in and recorded their laboratory meetings, accessed their grant proposals, papers, and laboratory books for a full year, to study their scientific thinking, reasoning, and discovery in real time ^101–103^.

The methodology used by Dunbar was considered novel in cognitive science studies. Patel and colleagues published a series on in vitro and in vivo studies of scientific reasoning in clinical setting and their relation to the nature of the errors generated. All these studies were included in a 2014 textbook ^104^. When investigating scientific thinking, reasoning, and discovery, such methodology and study setting provide the closest scenario and possibility to identify the process by which scientists make novel discoveries in real time. The results obtained through such a study can be incomparable. However, besides the obvious high costs of such a study, it is difficult to replicate and to scale up, considering the challenging criteria to meet for the investigation team who could conduct such studies and analyze the data collected as well as the candidate laboratories to choose from. Nevertheless, the results obtained are important and can be better than those experimental or simulated setting studies, i.e., in vitro studies. From his study, Dunbar concluded that for scientific discoveries to occur, analogies are critical, the research team should include different but overlapping scientific backgrounds, the projects attempted should include both high and low risks, that acknowledging and exploring further on unexpected results is crucial, and that the interaction among team members is essential ^101,102^. Among all the studies we reviewed in this paper, Dunbar’s study is the closest to our own, and for that reason, we organized the material and summarized side-by-side comparisons between the two studies in Table 1.

#### 2.4.2 What We Can Learn from the Literature

The field studies provide the best approach to study scientific hypothesis generation, problem-solving, results analysis, and scientific discovery in the real world and real-time directly; however, they are time-consuming and labor intensive and requires highly qualified investigators to conduct the study, to participate in, and to shadow. They are also difficult to repeat and scale up, and the study cycle is long. Despite these challenges the results obtained exceed those of any laboratory setting experiment. With acknowledgement the advantages of in vivo studies, our in vitro study has some strengths too, such as mimicking the real process, and ability to obtain data within a shortened timeframe, which makes the study more manageable and easier to operationalize.

### 2.5 LITERATURE SUMMARY

Studies on scientific thinking center their efforts on scientific reasoning and use scientific teaching and learning in school or university settings. Without diminishing the value of results obtained from such settings, we have shown in this review that those studies do not represent hypothesis generation in a scientific research context. To date, the literature lacks original in vitro studies. The in vivo cognitive study by Dunbar is a unique example, and this original study focused on scientific thinking and discovery in a scientific research context, provided an excellent method to study scientific thinking and discovery. The study, however, is difficult to replicate. In addition, other studies centered on medical education and aiming to train medical students into medical experts in clinical practice, did not incorporate a clinical research context. Despite these limitations, medical reasoning studies’ results and research methods have helped us formulate our research question and design our study significantly. Literature-based discovery studies, many of which used the ABC model as the conceptual framework, attempted to develop systems to facilitate users to generate hypotheses for their research studies. However, most studies in literature-based discovery did not conduct adequate human participant evaluations to provide direct evidence about the systems.

Although there are missing pieces in the literature related to scientific hypothesis generation, we emphasize the complementary nature of our work: we studied the scientific hypothesis generation process in clinical research contexts by leveraging findings and methodology from existing literature. Our study focused on exploring the process and mechanisms of the hypothesis generation process of clinical researchers and aiming to enlighten future tool development to facilitate this process and make it better. In other words, our work aimed to improve clinical research productivity and clinical research enterprise in the long term, which could be perceived as an extension of medical education but emphasized more on research capacity building and development. Therefore, our work has a slightly different end goal from the start. We do acknowledge that excellent progress has been achieved in scientific thinking, scientific and medical reasoning, and literature-based mining, all of which have provided the necessary foundation to initiate our work and make our exploration feasible on many levels.

### 2.6 OUR STUDY OBJECTIVES

We aimed to use this study to explore the role of VIADS during scientific hypothesis generation among clinical researchers. We aim to explore whether there are differences between experienced and inexperienced clinical researchers during scientific hypothesis generation because Patel et al. ^25,26,51,54^ demonstrated that there were differences among them during clinical reasoning for differential diagnosis. We summarize the methods and results in the next section to contextualize the perspectives shared in this article.

## 3. Review of Our Study

### 3.1 SUMMARY OF THE STUDY DESIGN AND METHODOLOGY

We conducted a 2 × 2 human participant study between August 2021 and November 2022 ^9^. We recruited clinical researchers and separated them into experienced and inexperienced groups based on predefined criteria ^10^. Then, within each group, participants were randomly assigned to the experimental or control groups. The experimental groups used VIADS as their analytical tool, and the control groups used other analytical tools, such as Excel, SAS, Stat, and SPSS, to analyze the same datasets in a maximum of 2-hour session. The datasets were derived from the National Ambulatory Medical Care Survey, i.e., NAMCS, conducted by the Centers for Disease Control and Prevention ^105^. We aggregated the International Classification of Diseases, Ninth Revision—ICD-9, codes from the surveys and included the most frequently used codes in 2005 and 2015 and the names of the ICD-9 codes in the data sets.

**Figure 2:**
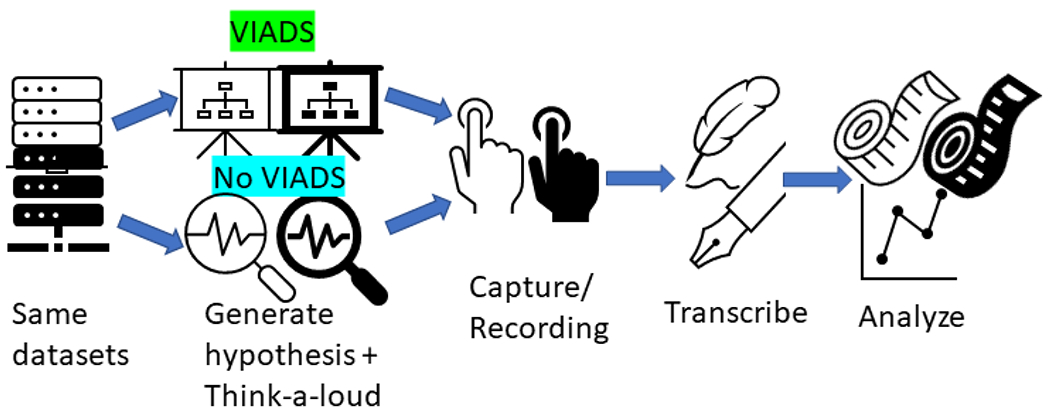
Summary of the data-driven hypothesis generation study flow

The VIADS groups had an additional one-hour training session to learn how to use VIADS. The participants were asked to conduct the data analysis and develop hypotheses using the think-aloud protocol to talk about what they are doing or intend to do in the process. All screen activities and audio during data analysis and hypothesis generation were recorded and transcribed by professional services for analysis. Participants were asked to complete surveys after the study sessions. The same study facilitator conducted all study sessions with each participant by following similar study scripts. The study protocol has been published ^10^. Figure 2 shows the general study flow.

Transcripts of the study session recordings were used to count the number of hypotheses generated by each participant. They were analyzed to measure the unit time required to generate each hypothesis on average. We also coded the transcription to identify the cognitive events used during hypothesis generation. We compared the results between the VIADS and control groups among inexperienced clinical researchers.

In order to compare the quality of hypotheses consistently, we developed and validated clinical research hypothesis evaluation metrics in a brief version, which includes significance, validity, and feasibility, and a comprehensive version which includes additional dimensions: novelty, clinical relevance, clarity, testability, potential benefits and risks, ethicality, and interesting. All hypotheses generated by the participants were assessed by an expert panel of seven members based on the same metrics. A detailed description of the metrics development, validation, and testing can be found in these references ^6,106^.

This study was approved by the Clemson University Institutional Review Board (IRB2020-056) and Ohio University Institutional Review Board (18-X-192). The invitation to participate was shared via national forums, such as the American Medical Informatics Association working groups, and international conferences, such as the European Federation for Medical Informatics, i.e., MIE 2022 ^107^, and by all research team members who reached out to their professional circles.

### 3.2 SUMMARY OF MAIN RESULTS

Fifteen inexperienced clinical researchers, including eight in the VIADS group and seven in the control group, and three experienced clinical researchers, including two in the VIADS group and one in the control group, completed the study during our study period. Experienced clinical researchers were underrepresented; therefore, their data were used for informational purposes without statistical analysis. Two additional clinical researchers, including one experienced and one inexperienced, participated in the pilot study to help finalize the study flow, scripts, and follow-up surveys before our formal study started. Detailed results can be found in the reference ^9^.

Clinical researchers generated 5–21 hypotheses, irrespective of quality. The VIADS group generated a similar number of hypotheses as the control group. Based on the same criteria, inexperienced clinical researchers had a valid rate of 63%, whereas experienced clinical researchers had a valid rate of 72%, more detailed results can be referred to the references ^7–9^.

The VIADS group required a statistically significantly shorter time than the control group to generate a hypothesis on average, i.e., 258 versus 379 seconds per hypothesis. The results were similar regardless of the categories of hypothesis, such as considering only valid or all hypotheses, including only inexperienced clinical researchers, or aggregating inexperienced and experienced clinical researchers. The VIADS group used significantly fewer cognitive events to generate each hypothesis on average, i.e., 4.48/hypothesis versus 7.38/hypothesis, which explained and supported the shorter time used by the VIADS participants. Moreover, the VIADS group had a much smaller standard deviation than the control group regarding the cognitive events used, i.e., 2.43 versus 5.02. More detailed results can be found in the references ^9,108^.

The expert panel used the brief version of the instrument to assess the quality of the hypotheses after reliability tests of both the brief and comprehensive versions of the instruments. The VIADS group received a slightly lower rating for significance and validity and a statistically significantly lower rating for feasibility regardless of the categories of hypotheses, that is, considering valid or all hypotheses, including inexperienced clinical researchers only or both. The feasibility ratings likely led to statistically significantly lower ratings when we combined the significance, validity, and feasibility ratings in the VIADS group. Meanwhile, we did notice VIADS groups generated more complex hypotheses than control groups, however, the complexity is not a measurable dimension in our current instruments. Detailed results can be found in the reference ^9^.

Our follow-up questions focused on participants’ past experiences related to hypothesis generation. Reading, conversations, and interactions with peers, colleagues, and advisors, as well as attending conferences were highly rated and repeatedly mentioned as events that had facilitated or provoked new ideas in the past. From the answers, we were unable to identify a single specific tool that could be used to facilitate the process or capture the initial ideas during the hypothesis generation process. Detailed results can be found in the references ^8,9^.

**Figure 3:**
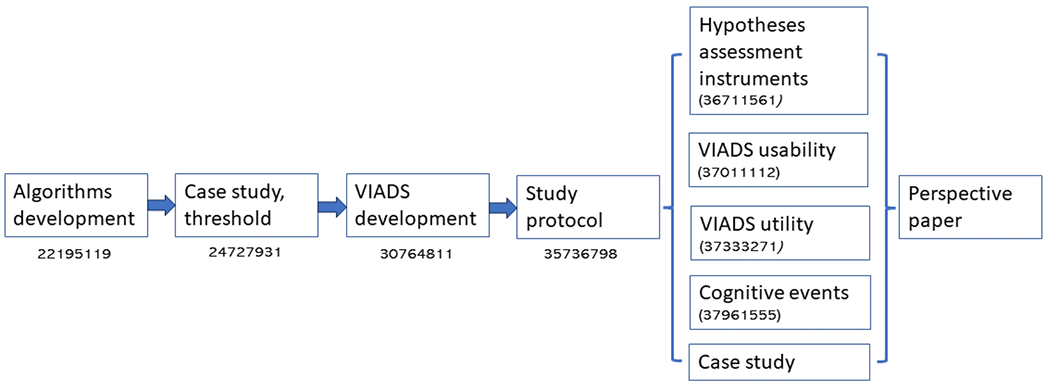
Summary flow of hypothesis generation project milestones and publications—# refers to PMID

The usability evaluation of VIADS was embedded in the hypothesis generation study sessions. The VIADS group participants were asked to complete an additional modified version of the System Usability Scale—SUS ^109,110^ survey in addition to the follow-up questionnaire at the end of their study sessions. The SUS score ranged widely, 37.5–87.5, with mean and median values of 71.9 and 75, respectively. Although the SUS score had a relatively large range, the participants provided overwhelmingly positive feedback on VIADS and unanimously agreed that VIADS offers new perspectives on datasets, see detailed results in the references ^8,9,107,111^. Figure 3 shows the summary milestones and publications of the project, and readers can refer to them for detailed descriptions of the respective methods and results.

### 3.3 DID WE ACCOMPLISH OUR STUDY OBJECTIVES?

The number of participants in the experienced group was insufficient to completely achieve our planned objectives. However, we obtained novel findings from the component that conducted among inexperienced clinical researchers. These findings are related to the baseline measurements, number, and mean unit time and cognitive events needed to generate a data-driven scientific hypothesis on average; and differences between inexperienced clinical researchers using VIADS or other analytical tools. Our results suggest that use of VIADS results in significantly shorter unit time and significantly fewer cognitive events to generate a hypothesis on average during the process. In addition, the use of VIADS scored significantly lower feasibility ratings than the control group who used other analytical tools. We also observed differences between experienced and inexperienced clinical researchers in their valid hypothesis rates when they were measured under the same standards and assessed by the same group of experts. The experienced group had 10% higher valid rate than the inexperienced clinical researcher group. In conclusion, although we could not completely answer the research questions raised at the beginning of the study, we are extremely encouraged by these novel findings, which provide us with adequate evidence to move the project to the next phase.

## 4. Discussion

### 4.1 RESULT INTERPRETATIONS AND SIGNIFICANCE

To the best of our knowledge, this is the first human participant study to generate data-driven scientific hypotheses of clinical research in a simulated setting. This work is significant for the following reasons. **First**, our experiments demonstrated the feasibility of the human participant study in capturing the hypothesis generation process in a clinical research context facilitated by data analytical tools and established the baseline measures. It also brought forth the fact that it is a truly challenging process. **Second**, our findings indicated that using VIADS improved the efficiency of the process among junior clinical researchers. We speculate that VIADS may have provided more structured guidance for clinical researchers during the hypothesis generation process, an explanation supported by the evidence from the comparison of the unit time per hypothesis and the cognitive events used between the VIADS and control groups. **Third**, we found that the VIADS group received a significantly lower rating in feasibility and subsequently in the total rating of the summation of feasibility, validity, and significance. We recognize that lower feasibility does not necessarily mean the participants in the VIADS group were more creative. However, the lower feasibility rating appeared to indicate a deviation in that direction. One likely scenario is that the participants in the VIADS group may have started to think in a more complex manner instead of linearly by looking at the hierarchical graphs generated by VIADS during the data analysis and hypothesis generation. These hierarchical graphs include not only hierarchies but also semantics. Additional rigorous and larger-scale studies will be required to prove this scenario. **Fourth**, the slightly lower ratings in validity and significance may be related to the one-hour training that the experimental group participants received to learn how to use VIADS. Six out of eight participants had a three-hour session with a brief break in between while the control group participants have a two-hour session. We wonder whether the three-hour session affected participants’ cognitive load negatively and unconsciously, since the hypothesis quality ratings indicated cognitive overload in the experimental group compared to the control group. Although the participants’ answers to the open-ended questions at the end of the study were positive about VIADS and its ability to present the data in new ways, the literature suggests that cognitive overload will negatively affect participants’ performance ^45^, especially when the participants learn a new tool and perform other tasks simultaneously. **Fifth**, we established metrics and instruments to measure scientific hypotheses in the clinical research context. The metrics and instruments are critical tools for consistently measuring hypotheses. They can also be used by peer reviewers during paper or grant proposal reviews or by investigators to prioritize multiple potential research projects before investing too much time and resources.

### 4.2 INSIGHTS, EXPERIENCE, AND LESSONS FOR FUTURE STUDIES

The literature, especially medical reasoning literature, indicates that the experience level is critical during medical reasoning. Experienced physicians and junior physicians use different strategies to solve clinical problems. For this reason, in our study design, we categorized clinical researchers into experienced and inexperienced groups based on their years of clinical research experience. We expected to determine whether similar differences exist among clinical researchers during hypothesis generation for research projects. However, although we used the same platforms and channels to recruit experienced and inexperienced clinical researchers, the recruitment efforts were unsuccessful among experienced clinical researchers and the experienced groups were underpowered. That component of our study did not generate anticipated results; the data collected were used for informational purposes without further statistical analysis. Experienced clinical researchers may have other priorities, and participating in a study on hypothesis generation may be outside their interests. However, experience in clinical research does not necessarily imply rigorous thinking, and observations suggest that some clinical researchers would still benefit from such activities or ways of thinking during hypothesis generation for research projects.

VIADS appears to be a helpful tool in secondary data analytical, summarizing, and visualization work, therefore enabling clinical researchers to generate hypotheses more efficiently. However, because of the complex nature of VIADS, we still need to elucidate which parts of VIADS play which role in facilitating hypothesis generation. For example, our current results cannot answer whether the visualization part of VIADS, the data analysis part of VIADS, or both worked in facilitating clinical researchers during hypothesis generation. In addition, VIADS, or the visualization parts, may stimulate participants’ thinking, as exemplified by the significantly lower feasibility ratings in the VIADS group. However, without a carefully designed study, we are uncertain of the speculation.

A few lessons learned during the study could be beneficial for future studies. We learned that it was critical to check the devices each time before a study session, more so when a new device or a new piece of the device was introduced, as we had to make sure that it was working with all existing software packages. We also realized the need to intentionally design the schedule to avoid a 3-hour continued session, as well as to separate the training and study sessions on different dates whenever possible. Alternatively, at the very least to separate the training and study sessions with a significant break in between, i.e., 5-10 minutes are inadequate. Although putting the two sessions together might be easier or more convenient for both participants and the study facilitator, the training and study sessions together can cause additional cognitive loads to participants, affecting the results negatively.

### 4.3 LIMITATIONS OF THE STUDY

Considering the complex nature of scientific hypothesis generation, many of the limitations of this study may be still beyond our current technological boundary. That is, some of the measurements may be beneficial in answering critical questions but unrealistic. For example, how exactly the scientific hypotheses were initiated and formed while participants analyzed datasets cannot be answered clearly because our current technology cannot yet capture the process explicitly. The think-aloud protocol is currently the only available method to capture the process; while it is not ideal, it is nonetheless a reality that we can use.

One of the study’s main limitations is the inadequate number of experienced clinical researchers, which prevented us from exploring the role of experience level during scientific hypothesis generation in clinical research. On the positive side, this may indicate that inexperienced clinical researchers are more eager to participate and could be future target users for any tools we develop for hypothesis generation. Meanwhile, this reality may indicate that experienced clinicians need more motivational encouragement and people skills to recruit successfully.

Furthermore, we do recognize this study’s limitations in capturing the hypothesis generation process. The think-aloud protocols have been a brilliant method in cognitive and psychology studies and usability testing ever since they were introduced by Ericson and Simon ^55^. Although we recognize that this is the best strategy, we could use to capture the hypothesis generation process, the approach is not perfect, and there are limitations. The think-aloud protocol can only capture conscious processes articulated by participants. Therefore, we could say that our study revealed part of the process, not yet the whole cognitive process. It is potentially impossible to reveal the complete cognitive process of scientific hypothesis generation with our available technologies and approaches, a challenge beyond our current capacity.

The last limitation is related to VIADS, which is a secondary data analytical tool by nature. Although it can facilitate hypothesis generation, it was not explicitly designed for this purpose. Although VIADS still shows its effectiveness in facilitating inexperienced clinical researchers in generating hypotheses, we believe that a more comprehensive tool to specifically support hypothesis generation will be much more effective.

### 4.4 OPPORTUNITIES FOR FUTURE STUDIES

The first opportunity is to capture participant’s thinking process more completely and accurately, which may include scientific hypothesis generation, scientific thinking, or scientific reasoning. With a better understanding of the thinking process, the results can be translated to guide the design and development of corresponding tools to improve the process. This means understanding how scientist think, and there are several studies on this topic. Another opportunity is the lack of support for scientific hypothesis generation. From the answers to our open-ended questions at the end of the study sessions and our own experience, there appear to be no specific tools to support the process. Considering the emergence of large language models, a probability model with an exceptional capability to predict and generate human-like fluent language, it reminded us that hypothesis generation is perhaps one of the unique traits of the human brain. However, we have an extremely limited understanding of the process, not to mention how to facilitate it to make it better. The area is unique and critical enough to be studied further and more thoroughly to maintain the strengths of the human species and improve research productivity and output overall.

## 5. Conclusion

Hypothesis generation is an important first step in any scientific research. It is difficult to exemplify the process in concrete ways; therefore, it is difficult to teach and reproduce, even for successful investigation teams, investigators, and discoveries. However, it is a critical and early stage of the clinical research project life cycle. The more we understand the process, the better we may be able to facilitate and improve it, the clinical research projects, and the clinical research enterprise as a whole. From our human subject study, we have learned that intentional and structured guidance during hypothesis generation can facilitate the process, at least among inexperienced clinical researchers. VIADS, as an example of a potential tool, appears to make the hypothesis generation process more efficient, that is, significantly faster, by using significantly fewer cognitive events. Meanwhile, the number of hypotheses generated was similar between the VIADS and control groups. Regarding the quality of the hypotheses, the control group was slightly higher in validity, significance, and the feasibility is statistically significantly higher. We do notice the hypotheses generated by the VIADS groups seemed more complicated than those generate by the control groups. Therefore, we noted the results as mixed and inconclusive as to whether VIADS is helpful in the hypothesis generation process. The role of VIADS in hypothesis generation may be more complicated than that of linear effects. A larger-scale study with more functional tools focusing on hypothesis generation would likely generate more generalizable results, considering that VIADS is a secondary data analytical tool that was not developed primarily to facilitate hypothesis generation.

## Figures and Tables

**Table 1:** Comparison of Dunbar’s in vivo cognitive study ^101,102^ and our in vitro scientific hypothesis generation study in clinical research ^7–10^

Dimensions	In vivo scientific thinking	In vitro hypothesis generation in clinical research
**Study setting**	Field study	Simulated/experimental setting
**Subjects**	4 laboratories, 19 scientists	20 clinical researchers
**Study timeframe**	1 year	2–3 hours/person
**Investigator**	Same person	Same person
**Datasets**	What they are working on	Same datasets for all
**Subject activities**	Regular scientific/daily work	Analyze data and develop hypotheses
**Purposes**	Decipher scientific thinking and discovery naturally	Identify clinical researchers’ data-driven hypotheses generation process
**Data collection**	Interviews	Recording screen activities
	Laboratory meetings (recording)	Recording audio (think-aloud)
	Access to data, laboratory notes, proposals, and papers	Follow-up surveys
**Data analysis**	Analyze and categorize recordings, laboratory notes, and observations	Analyze recordings, assess hypotheses, time, count, and hypothesis quality comparison
**Results**	Analogy, backgrounds of laboratory members, high-and low-risk projects, unexpected results, and interactions among members	Number of hypotheses/person, time/hypothesis, hypothesis quality assessment instruments, hypothesis quality ratings and comparisons, and cognitive events/hypothesis
